# Development of *Saccharomyces cerevisiae* isobutanol production strain from osmotolerant and ethanol-producing industrial isolated yeast

**DOI:** 10.1016/j.btre.2026.e00959

**Published:** 2026-04-22

**Authors:** Naphattarachon Thammapanyaphong, Manutsanun Boonyanuwat, Apanee Luengnaruemitchai, Jirasin Koonthongkaew

**Affiliations:** a Department of Microbiology, Faculty of Sciences, Chulalongkorn University, Phayathai Rd., Pathumwan, Bangkok 10330, Thailand; b Research Unit in Bioconversion/Bioseperation for Value-Added Chemical Production, Chulalongkorn University, Bangkok 10330, Thailand; c The Petroleum and Petrochemical College, Chulalongkorn University, Bangkok 10330, Thailand; d Center of Excellence on Catalysis for Bioenergy and Renewable Chemicals (CBRC), Chulalongkorn University, Bangkok 10330, Thailand

**Keywords:** *Saccharomyces cerevisiae*, Isobutanol, Isobutanol toxicity tolerant, Mitochondrial Branched-chain amino acid aminotransferase Bat1, CRISPR/Cas9

## Abstract

Isobutanol is a promising advanced biofuel alternative to ethanol for advanced biofuels and can be converted into sustainable aviation fuel (SAF) via the alcohol-to-jet (ATJ) process. Recent studies attempted to improve isobutanol production from *S. cerevisiae* by metabolic engineering; nevertheless, engineered strains, mostly derived from laboratory strains, often encountered difficulties in industrial-scale production. Herein, this present study aims to develop an industrially viable isobutanol-producing strain of *S. cerevisiae* D3C (isolate G2–3–2, identified in the previous study) with minimal genetic modification. Isolate G2–3–2 was subjected to conventional mutagenesis, resulting in the development of an isobutanol-tolerant strain, IbOH-1. The genomic DNA analysis of IbOH-1 revealed numerous mutations in genes associated with nitrogen starvation response, cell wall biosynthesis and integrity, stress resistance-related amino acid biosynthesis, and the HOG pathway, all of which may collectively enhance tolerance to isobutanol toxicity. The *BAT1* gene was afterwards knocked out by CRISPR/Cas9 to improve isobutanol yield. The isobutanol production from IbOH-1*bat1∆* strain was investigated in both nutrient-rich YPD and minimal YNB fermentation media, with particular emphasis on optimizing glucose concentration. The highest concentration of isobutanol attained was 3.12 ± 0.068 g/L in 5-L bioreactor (with 3 L working volume), utilizing YNB based medium containing 150 g/L of glucose, supplemented with 8 g/L of peptone, 3 g/L of (NH_4_)_2_SO_4_, 1 g/L of KH_2_PO_4_, 0.5 g/L of MgSO_4_·7H_2_O, and 0.05 g/L of FeSO_4_·7H_2_O. This study provides new insights into improving isobutanol production in *S. cerevisiae*, highlighting its potential applicability in industrial biofuel processes.

## Highlights

• Isobutanol-producing *S. cerevisiae* strain for industrial application.
• Isobutanol-tolerant strain developed from conventional mutagenesis.
• CRISPR-Cas9-mediated BAT1 knockout to enhance isobutanol production.
• CRISPR-Cas9 avoids the presence of plasmids and antibiotic markers.
• Strain highly increased isobutanol yield, potential for bio-alcohol production.

## Graphical abstract

**Figure:**
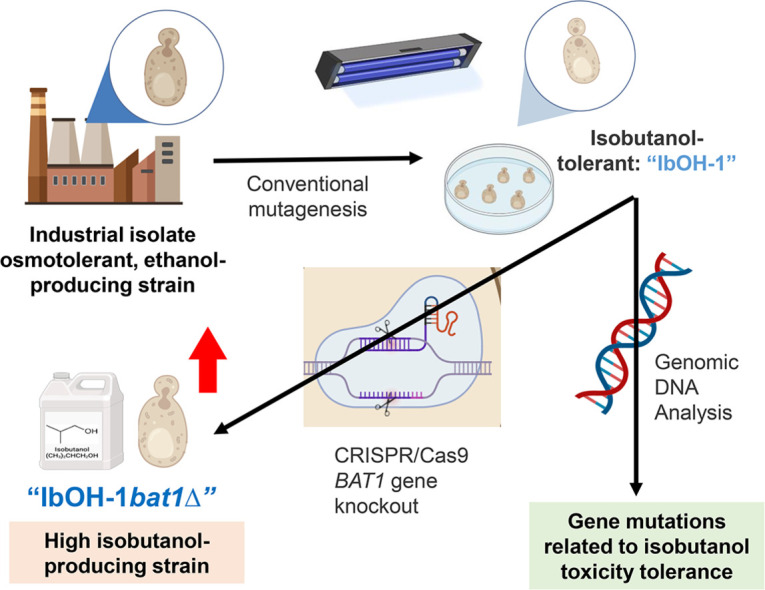

## 1 Introduction

Isobutanol (IUPAC: 2-methylpropan-1-ol) is classified as branched-chain higher alcohol (BCHA), characterized as an aliphatic alcohol with a branching structure, with a greater number of carbon atoms, molecular weight, and boiling point than ethanol. This compound is frequently utilized in several chemical industries, including solvents, coatings, and chemical intermediates, as well as in petrochemical industries as a precursor for polymer synthesis through the dehydrated derivative of isobutanol: isobutene or isobutylene [1]. Recent studies have revealed that isobutanol serves as a superior liquid biofuel to replace bioethanol, owing to its superior combustibility [[2], [3], [4]]. Moreover, isobutanol is a preferred alcohol-based substrate for producing sustainable aviation fuel (SAF) via the alcohol-to-jet (ATJ) process. The dehydrated olefin form of isobutanol, known as isobutene or isobutylene, undergoes oligomerization into C12–16 higher olefins and is then hydrogenated into long-chain alkanes, which constitute petroleum jet fuel [5]. Notably, such ATJ fuels can be blended with regular petroleum jet fuel at a ratio of up to 50% (v/v), hence potentially diminishing carbon dioxide (CO_2_) emissions from the aviation sector [5]. In light of the legislative requirement to diminish CO_2_ emissions from the aviation sector and the extensive benefits across various industries, the global market of isobutanol is projected to increase from US$ 833.3 million in 2022 to US$ 1.3 billion by the conclusion of 2031 (estimated to grow at a CAGR of 5.2% from 2023 to 2031) [Isobutanol Market Size, Growth, Industry Share -2031 (transparencymarketresearch.com), accessed on 13 June 2025].

**Fig. 1:**
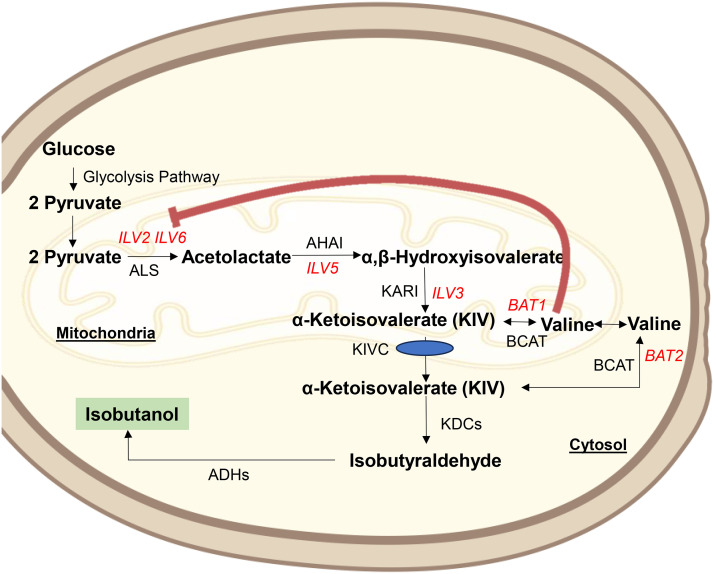
Schematic representation of the isobutanol production pathway in the yeast *Saccharomyces cerevisiae*. KIV, the key intermediate, follows the isobutanol production route by being transported into the cytosol using the unidentified mitochondrial KIV carrier (KIVC), where it is further converted to isobutanol by KDCs and ADHs, respectively. This isobutanol production route, which requires the transport of KIV via KIVC, was designated the KIVC-dependent pathway. The red arrow illustrates feedback inhibition of valine to the regulatory subunit of Acetohydroxyacid Synthase, Ilv6.

Isobutanol is traditionally synthesized through petrochemical processes, primarily via the carbonylation of propylene, hydroformylation, and Reppe carbonylation reactions [6]. Those traditional approaches are neither sustainable nor environmentally benign [1]. Consequently, the biosynthesis of isobutanol from microorganisms is more appealing due to its environmental sustainability, reduced energy usage, and superior efficiency compared to the petrochemical method [7]. Among all microorganisms, the yeast *Saccharomyces cerevisiae* gives greater advantages over bacteria as a host for isobutanol production due to its superior isobutanol tolerance, its ability to naturally synthesize small quantities of isobutanol as a by-product of fermentation, and ensuring bioprocess stability against bacteriophage contamination in large-scale industrial fermentation [8]. Furthermore, possessing Generally Recognized as Safe (GRAS) certification for consuming, *S. cerevisiae* is conventionally utilized in various industries, including fermentation and brewing [9]. *S. cerevisiae* produces isobutanol via the Ehrlich degradation pathway of the branched-chain amino acid valine (Val) through important intermediates: α-ketoisovalerate (KIV) [10]. (see also, Fig. 1). In the natural process, pyruvates are subsequent to transport into the mitochondria, two pyruvate molecules are condensed into one molecule of acetolactate by acetohydroxyacid synthase [AHAS (Ilv2/Ilv6), with the catalytic subunit Ilv2 (encoded by the *ILV2* gene) and the regulatory subunit Ilv6 (encoded by the *ILV6* gene)]. Subsequently, acetolactate is converted into α, β-dihydroxyisovalerate and α-ketoisovalerate (KIV) by the activity of acetohydroxyacid isomerase (Ilv5, encoded by the *ILV5* gene) and acetohydroxyacid dehydratase (Ilv3, represented by the *ILV3* gene). KIV, the key intermediate, is either transformed into valine in mitochondria by branched-chain amino acid aminotransferase (BCAT): Bat1 (encoded by the *BAT1* gene) or translocated into the cytosol. KIV is also synthesized in cytosol by the Ehrlich degradation of the amino acid valine (Val), commencing with a transamination conversion of Val to KIV mediated by cytosolic BCAT: Bat2, which is encoded by the *BAT2* gene. KIV undergoes further decarboxylation to form isobutyraldehyde via α-keto acid decarboxylases (KDCs). Ultimately, isobutyraldehyde undergoes reduction to isobutanol facilitated by alcohol dehydrogenases (ADHs) [11]. (Fig. 1).

The industrial produce of isobutanol presently encounters numerous challenges. One of the primary limitations is the low yield of isobutanol in the native *S. cerevisiae* strain [12]. Owing to the inherently low isobutanol yield in *S. cerevisiae,* several recent studies aimed to enhance isobutanol production from *S. cerevisiae* by metabolic engineering [3,8,13], mitochondrial compartmentalization of isobutanol biosynthesis pathways [14], cofactor balancing [15], and bottleneck-enzyme (Bat1 and Bat2) engineering [4]. Nevertheless, the toxicity of isobutanol remains another significant factor that constrains production [16]. Isobutanol exhibits more toxicity to yeast cells than ethanol, attributable to the disruption of plasma membrane lipids, membrane transport mechanisms, and the initiation of protein translation [[17], [18], [19]]. A recent study aimed to enhance resistance to isobutanol toxicity; nonetheless, the productivity of isobutanol remains significantly below industrial viability [16]. One hypothesis pertains to the origin of the isobutanol producing strain. Recent studies developed isobutanol-producing strains from laboratory strains, which encounter hurdles when scaled for industrial bioprocessing [20]. Moreover, in industrial bioprocessing, designed microbes from recombinant DNA technology demonstrate notable shortcomings, such as restricted tolerance to diverse stresses, substrate specificity, creation of undesired byproducts, and sluggish growth rates [21]. No industrially viable isobutanol producing strain of *S. cerevisiae* has been established to date.

Hence, this study aims to develop an appropriate *S. cerevisiae* strain for industrial isobutanol production. The yeast strain utilized in this study is isolate G2–3–2 (*S. cerevisiae* strain D3C), sourced from the Thai sugar industry: Thai Multi-Sugar Industry, as reported in a previous study [22]. Isolate G2–3–2 has been identified as an osmotolerant strain with tremendous efficacy in ethanol production, which could potentially be utilized in the industrial production of bio-alcohols. The toxicity of isobutanol to the growing strain was eradicated using conventional mutagenesis using ultraviolet (UV) irradiation, a method acknowledged as effective across several industries [23], followed by adaptive acclimatization for isobutanol tolerance. The isobutanol-tolerant strain was further modified to enhance isobutanol production by knocking out *BAT1*, a rate-limiting enzyme and competitive pathway for isobutanol synthesis, employing the CRISPR/Cas9 gene editing technique while circumventing the introduction of cloning vectors and antibiotic selectable markers in the isobutanol-producing strain [3,4]. Simultaneously, the mechanisms of isobutanol tolerance in the isobutanol-tolerant strain were preliminarily investigated and anticipated using whole-genomic DNA sequencing.

## 2 Materials and methods

### 2.1 Yeast strains and culture media

The osmotolerant strain of *Saccharomyces cerevisiae* (G2–3–2, *S. cerevisiae* strain D3C) from the previous study [22]. was used as a parental strain for conventional mutagenesis. Yeast cells were cultured in nutrient-rich yeast extract-peptone-dextrose (YPD) medium (10 g/L yeast extract, 20 g/L peptone, and 20 g/L glucose) and nitrogen base (YNB) medium (1.5 g/L bacto yeast nitrogen and ammonium sulfate, 5 g/L ammonium sulfate, 20 g/L glucose) as fermentation media for isobutanol production. All media were solidified by supplementation with 2% agar in case it is necessary.

### 2.2 Isolation of isobutanol-tolerant mutants

Conventional mutagenesis by ultraviolet (UV) irradiation was conducted following a previously described protocol, with minor modifications [24]. Yeast cells were pre-cultured in 5 mL of YPD medium at 30 °C for 18 h. The culture was then adjusted to a density of approximately 5 × 10^7^ cells/mL. UV mutagenesis was performed using a 254 nm UV lamp at a distance of 25 cm for 2–4 min. The irradiated cells were cultivated in YPD agar and incubated at 30 °C for 2 days to allow colony development.

Subsequently, the cells were cultured in YPD medium supplemented with increasing concentrations of isobutanol to promote acclimation to isobutanol stress modified from the previous study [25]. Briefly, 1 mL of cultured cell suspension was transferred every 24 h into fresh YPD medium containing progressively higher isobutanol concentrations, ranging from 12 to 21 g/L. Once the cells successfully adapted to 21 g/L isobutanol, the cultures were plated onto YPD agar. Colonies obtained from these plates were selected as candidate mutants for further evaluation of isobutanol tolerance.

To evaluate isobutanol tolerance, a viable plate count assay was performed based on a previously described method with modifications [16]. Briefly, the yeast cells were pre-cultured in YPD media at 30 °C for 18 h. The cell suspensions were then adjusted to an initial OD_600_ = 1 and transferred to YPD medium supplemented with 21 g/L isobutanol, followed by incubation at 30 °C with shaking at 200 rpm. Every 24 h, 0.1 mL of the cell suspension was collected, diluted to an appropriate dilution, and spread on a YPD agar plate. The YPD plates were incubated at 30 °C for 2–3 days to calculate colony-forming units (CFU/mL) and % viability, respectively.

### 2.3 Examination of osmotolerant phenotype

The osmotolerant phenotype of the candidate isobutanol-tolerant mutant was confirmed using a similar method from a previous study with some modifications [22]. The cells were pre-cultured in YPD media at 30 °C for 18 h. Then, the cell suspensions were transferred to YPD containing 300 g/L of glucose. OD_600_ was measured every 24 h until reaching 3 days to examine the osmotolerance under high-glucose conditions.

### 2.4 Whole genome sequencing analysis

The IbOH-1 strain (isobutanol-tolerant strain) from the previous section was submitted to perform whole genome DNA sequencing to identify the *BAT1* gene sequence and key mutations responsible for tolerance mechanisms to isobutanol toxicity comparing to the parental strain G2–3–2. Those two yeast strains were submitted to GIBTHAI COMPANY (Thailand) and forwarded to Macrogen Company (Korea) for DNA extraction and library construction. Genomic DNA from the parental strain G2–3–2 was extracted and subjected to whole genome sequencing using Illumina technology. De novo assembly was performed to construct a reference genome library for G2–3–2. Genomic DNA from the mutant strain IbOH-1 was then sequenced and analyzed by resequencing against the G2–3–2 reference to identify genomic variations associated with isobutanol tolerance.

All statistical and bioinformatic analyses were performed by the company using the following programs: FastQC, Trimmomatic, Jellyfish, Genomescope, Platanus-allee, Busco, BLAST, MAKER (v3.01.03), and BLAST+(V.2.7.1+).

### 2.5 *BAT1* gene knocking out by CRISPR/Cas-based genome editing

The IbOH-1 mutant strain was further modified to enhance isobutanol productivity by knocking out the bottleneck enzyme, the *BAT1* gene [3]. The *BAT1* gene encodes the mitochondrial branched-chain aminotransferase (mBCAT), a rate-limiting enzyme in valine biosynthesis. Valine accumulation exerts feedback inhibition on *ILV6*, the regulatory subunit of acetohydroxyacid synthase (AHAS), which catalyzes the first committed step in the synthesis of α-ketoisovalerate (KIV), a key intermediate for isobutanol production [3,26]. To circumvent this limitation, the *BAT1* gene was disrupted using the CRISPR/Cas9 gene editing system. The isobutanol-tolerant mutant strain was submitted to Getz Healthcare (Thailand) and further processed to SYNBIO TECHNOLOGIES company (China) for disruption of the *BAT1* gene. The *BAT1* gene knockout was confirmed by the company via *BAT1* gene sequencing using the primers listed.

### 2.6 Fermentative production of isobutanol

High cell-density fermentation for isobutanol production was performed as described in the previous study with some modifications [3]. The yeast cells were pre-cultured in 5 mL of YPD medium at 30 °C for 18–24 h. On the following day, the cells were collected by centrifuging at 10,000 rpm for 15 min and resuspended in 10 mL of YNB medium supplemented with varying glucose concentrations (10–25%, equivalent to 100–250 g/L) to determine the optimal carbon source level for isobutanol production. After identifying the optimal glucose concentration, pH optimization was conducted by adjusting the medium to pH 4.0, 5.0, 6.0, or 7.0. The cultures were incubated at 30 °C with shaking at 200 rpm for 2–3 days. Subsequently, culture supernatants were collected and analyzed for isobutanol concentration.

The isobutanol production medium was modified by supplementing with additional nutrients, following adaptations from a previously reported protocol [27]. Briefly, yeast cells were pre-cultured in YPD medium containing 20 g/L of glucose at 30 °C with agitation at 200 rpm for 24 h. High cell density suspension was inoculated into the modified YNB-based media [supplemented with 8 g/L of peptone, 3 g/L of (NH_4_)_2_SO_4_, 1 g/L of KH_2_PO_4_, 0.5 g/L of MgSO_4_·7H_2_O, and 0.05 g/L of FeSO_4_·7H_2_O] with the previously optimized glucose concentration and pH value. The cultures were incubated at 30 °C with shaking at 200 rpm for 3 days. Following incubation, the cultures were centrifuged at 10,000 rpm to collect the supernatant for isobutanol quantification.

### 2.7 Fermentative production of isobutanol in 5-L bioreactor

The isobutanol fermentation procedure in a 5-L bioreactor was adapted from a previous study with slight modifications [28]. Briefly, the IbOH-1*bat1*Δ cells were pre-cultured in 10 mL of YPD broth at 30 °C and 200 rpm. The culture was then sequentially scaled up using a 10% (v/v) inoculum into 25, 50, 100, and 500 mL seed cultures, respectively. After 24 h of incubation, the cells were harvested by centrifugation at 10,000 rpm for 15 min, washed twice with sterile water, and collected as cell pellets. The cell pellets were then resuspended in 3 L of a YNB-based medium supplemented with 8 g/L peptone, 3 g/L (NH_4_)_2_SO_4_, 1 g/L KH_2_PO_4_, 0.5 g/L MgSO_4_·7H_2_O, and 0.05 g/L FeSO_4_·7H_2_O, containing 150 g/L glucose, with the pH adjusted to 7.0. Fermentation was carried out in a 5-L bioreactor with a working volume of 3 L at 30 °C, with an agitation speed of 250 rpm and an aeration rate of 1.5 vvm for 72 h. Every 4 h, samples were collected to measure glucose consumption, isobutanol concentration, and optical density at 600 nm using HPLC and a Genesys 30 ultraviolet-visible spectrophotometer (Thermo Scientific, NY), respectively.

### 2.8 Quantification of isobutanol content

The amount of isobutanol in the fermented broths from each trial was measured using high-performance liquid chromatography (HPLC, Agilent Technology, Model: 1260 Infinity II) under the conditions established by [29]. This setup included an Aminex HPX-87H column (300 mm × 78 mm, Bio-Rad Lab), a de-ashing cartridge holder (30 mm × 4.6 mm, Bio-Rad Lab), and a micro-guard cation H^+^ refill cartridge holder (30 mm × 4.6 mm, Bio-Rad Lab). Following the method described by Chinwatpaiboon et al. (2023), the column was eluted with 5 mM H_2_SO_4_ at a flow rate of 0.6 mL/min, and the column oven was maintained at 60 °C. A 50-μL sample volume was injected, and the analysis was detected using a refractive index detector (Agilent Technology, Model: 1260 RID) at 40 °C.

## 3 Results and discussion

### 3.1 Development of isobutanol-tolerant yeast strain

Despite the advantages of isobutanol in several industries, the production of isobutanol using the yeast *S. cerevisiae* confronts a significant bottleneck due to the higher toxicity of isobutanol compared to ethanol, which adversely affects yeast cell viability [30]. Previous study has shown that combining isobutanol tolerance with overexpression of genes involved in isobutanol biosynthesis can enhance production [16]. To address the toxicity challenge, we initially developed an isobutanol-tolerant strain of *S. cerevisiae* from a suitable osmotolerant ethanol-producing strain. The osmotolerant strain was derived from previous work; *S. cerevisiae* D3C (isolate G2–3–2) demonstrated tolerance to 280 g/L of glucose [22].

**Fig. 2:**
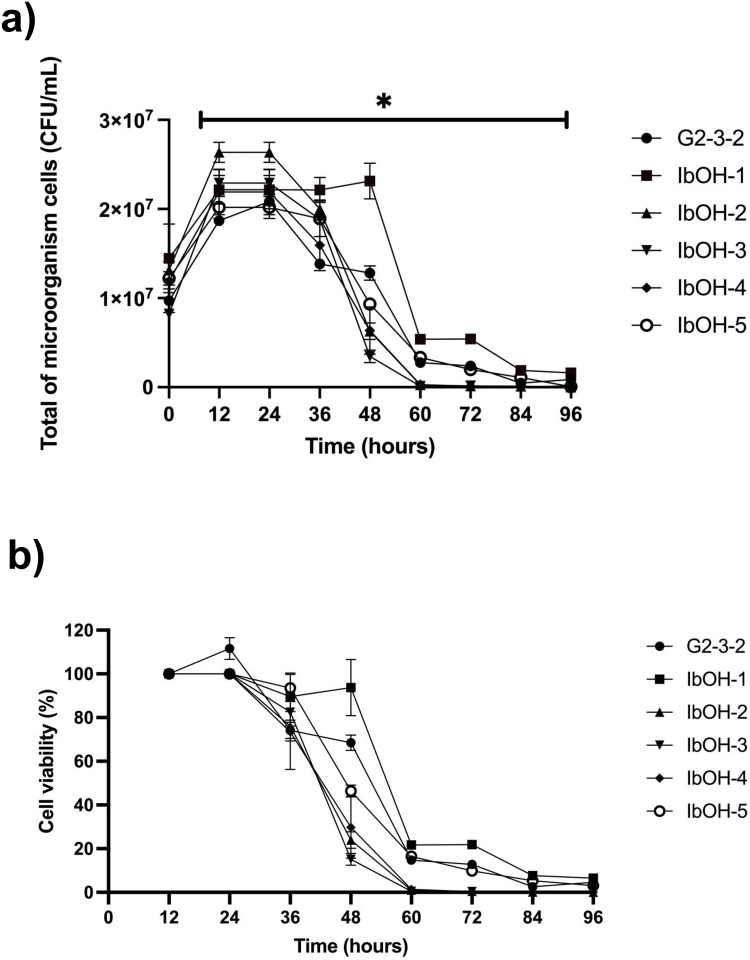
Cell viability as colony-forming units per milliliter (CFU/mL) (a) and percentage of cell viability (b) of parental strain G2–3–2 in comparison to mutant strains. Statistically significant differences among six strains were verified by two-way ANOVA followed by Tukey’s multiple comparisons test (*P < 0.05).

Following conventional mutagenesis using ultraviolet (UV) irradiation, a survival rate of ∼1.5–2% and subsequent acclimatization to isobutanol toxicity, five mutant colonies were isolated and designated as mutants IbOH-1, IbOH-2, IbOH-3, IbOH-4, and IbOH-5. Subsequently, the mutant strains were assessed for isobutanol tolerance in YPD media containing 21 g/L of isobutanol. The cell viability results were determined as colony-forming units per milliliter (CFU/mL) and percentage of cell viability, by comparing CFU/mL values from each time point with the 12-hour value (Fig. 2a and b). This concentration of 21 g/L isobutanol was selected as it represents a high-stress condition, well above the concentrations typically reported in previous studies. Adaptation experiments have generally been performed at ≤ 20 g/L isobutanol, while common laboratory strains of *S. cerevisiae* (BY4741 and CEN.PK113–7D) show strong growth inhibition above ∼1% (v/v) and no growth at ∼2% (v/v), corresponding to approximately ∼8–16 g/L of isobutanol, respectively. Thus, 21 g/L provides a selective pressure that exceeds the inhibitory thresholds of wild-type yeast and challenges only mutants with genuinely enhanced tolerance [16,28], thereby enabling discrimination between mutants with marginal and those with substantially enhanced tolerance. In comparison, the cell viability of the parental strain G2–3–2 declined after 24 h, with total viable cells measured at 1.3 × 10^7^ ± 8 × 10^5^ CFU/mL after 48 h, corresponding to approximately 60% of viability (Fig. 2a). Interestingly, under the same conditions, the mutant strain IbOH-1 maintained cell viability for up to 48 h, with total viable cells at 2.3 × 10^7^ ± 1.9 × 10^6^ CFU/mL, indicating nearly 100% viability at 48 h. The other mutants exhibited reduced isobutanol tolerance compared to G2–3–2, with cell viability rates at 48 h assessed being around 50%, 30%, 25%, and 20% for IbOH-5, IbOH-4, IbOH-2, and IbOH-3, respectively (Fig. 2b).

Additionally, the osmotolerant characteristic of IbOH-1 was confirmed (Table 1). When cultured in medium containing 300 g/L glucose, the optical density at 600 nm (OD_600_) of IbOH-1 reached 0.375 ± 0.005 per mL at 72 h, which was not significantly different from that of the parental strain G2–3–2 (OD_600_ = 0.384 ± 0.040 per mL at 72 h). Based on these results, IbOH-1 was selected as the isobutanol-tolerant strain for further studies.

**Table 1:** Osmotolerant phenotype of G2–3–2 original strain and IbOH-1 mutant. The cells were cultured in media containing 300 g/L of glucose. The values are the means and standard deviations of results from three independent experiments.

**Yeast Strains**	**Optical Density at 600nm (OD_600_)**		
	**24 h**	**48 h**	**72 h**
**G2–3–2**	0.327 ± 0.015N.S.	0.370 ± 0.042 N.S.	0.384 ± 0.040 N.S.
**IbOH-1**	0.280 ± 0.018 N.S.	0.340 ± 0.021 N.S.	0.375 ± 0.005 N.S.

N.S Indicates non-significant difference of OD_600_ values between G2–3–2 and IbOH1 at each time point (statistically verified by student’s *t*-test).

### 3.2 *BAT1* gene sequence identification

*S. cerevisiae* possesses several benefits compared to other microorganisms and is conventionally utilized for bio-alcohol production, particularly bioethanol [31]. Nonetheless, the wild-type strain of this yeast can produce isobutanol in small amounts (not exceeding 6.4 mg/L) due to the regulatory mechanisms controlling the isobutanol synthesis pathway linked to valine metabolism [8]. Recall, the rate-limiting and bottleneck enzyme that limits isobutanol productivity is branched-chain aminotransferases [BCATs: Bat1 and Bat2 (encoded by *BAT1* and *BAT2* genes)] (see also, Fig. 1) [32]. Hammer and Avalos (2017) demonstrated that deletion of the *BAT1* gene is the most effective approach to improve isobutanol productivity [3]. Thus, the *BAT1* gene has been identified as our target for knocking out to augment isobutanol productivity from the IbOH-1 mutant derived from the previous section.

**Fig. 3:**
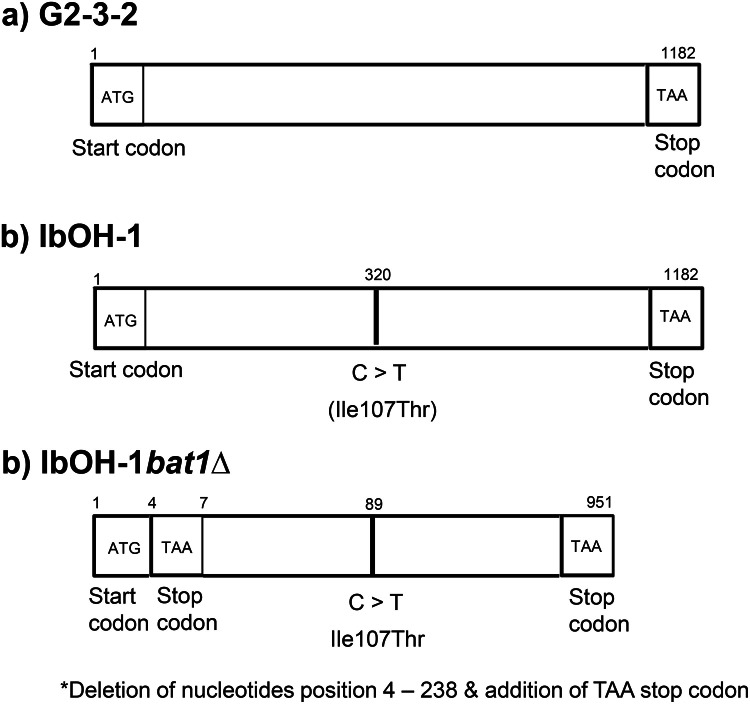
Schematic structure of *BAT1* gene in G2–3–2 (a), IbOH-1 (b), IbOH-1*bat1∆* (c).

**Fig. 4:**
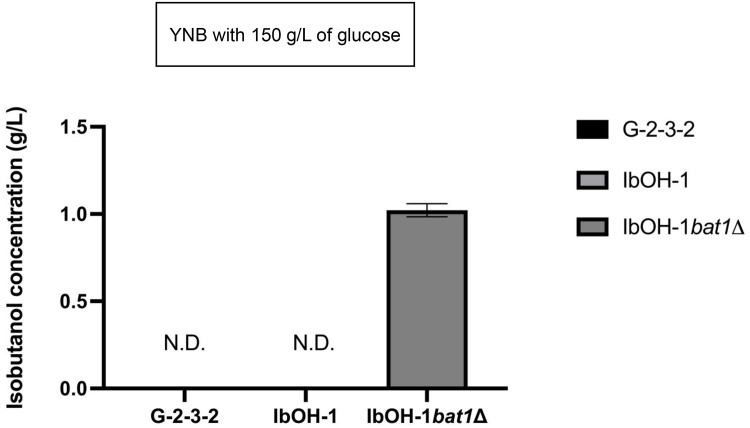
Isobutanol concentration (g/L) from G2–3–2, IbOH-1, and IbOH-1*bat1*∆ (^N.D.^indicates not-detected amount of isobutanol).

Genomic DNA sequencing data for the parental strain G2–3–2 were not reported in previous work [22]. Therefore, we initially conducted whole-genome DNA sequencing of G2–3–2 and IbOH-1 to identify mutations obtained from UV mutagenesis. Sequencing examination revealed one mutation in the *BAT1* gene of IbOH-1, wherein a cytosine (C) at nucleotide position 320 was replaced by thymine (T), resulting in an amino acid substitution from isoleucine (Ile) to threonine (Thr) at position 107 (Fig. 3a-3b and **Supplementary figure S1)**. To investigate whether the mutation in the *BAT1* gene of IbOH-1 affected Bat1 enzyme function, isobutanol content was compared between G2–3–2 and the IbOH-1. The results indicated that the isobutanol concentration in both strains was below the quantification limit (Fig. 4). The results indicated that additional *BAT1* gene disruption was necessary to improve isobutanol production from IbOH-1.

### 3.3 CRISPR-Cas9-mediated knockout of the *BAT1* gene

As previously mentioned, the disruption or inactivation of the rate-limiting enzyme Bat1, encoded by the *BAT1* gene, is essential for augmenting isobutanol synthesis in *S. cerevisiae* [3,4,33]. CRISPR-Cas9-mediated *BAT1* gene knockout, designated IbOH-1*bat1*Δ, was verified by the company through DNA sequencing of the *BAT1* locus (Fig. 3c and **Supplementary figure S2)**. The deletion of nucleotide positions 4–238 and the introduction of a stop codon (TAA) after the start codon (ATG) inhibits the functionality of the *BAT1* gene in IbOH-1*bat1*Δ **(Supplementary table S2)**. The *BAT1* gene in the IbOH-1*bat1*Δ mutant contained 951 nucleotides, compared to 1182 nucleotides in the wild-type *BAT1* gene of the IbOH-1 strain (Fig. 3b and 3c).

The CRISPR-Cas9 gene knockout technique offers a distinct advantage over traditional methods by eliminating the necessity for antibiotic resistance markers and cloning plasmids, which are unsuitable for industrial production [21]. The recombinant yeast containing the cloning plasmid and resistance marker frequently encounters instability issues with the cloning vector, resulting in elevated maintenance costs [34]. Following *BAT1* gene knockout, the cytosolic aminotransferase *BAT2* gene may assume a critical role in branched-chain amino acid metabolism. While Bat2 can partially compensate for the loss of Bat1, its cytosolic localization may alter the distribution of valine-derived intermediates, potentially influencing the flux toward isobutanol and other higher alcohols. In the IbOH-1*bat1∆*, Bat2 expression or activity may have been modulated, contributing to the observed tolerance and production phenotype. Similar effects have been reported for Bat1 loss-of-function, which reduces intracellular BCAA levels while increasing fusel alcohol formation, highlighting the interplay between mitochondrial and cytosolic aminotransferases in shaping metabolic flux [3,33]. Although these two branched-chain amino acid aminotransferases (BCATs) exhibit differences in localization and transcriptional regulation profiles, previous studies found that the relocalization of Bat2 into the mitochondria of bat1*∆* cells could rescue valine production [35,36]. This aligns with a recent study verifying the enzymatic activity of recombinant Bat1 and Bat2 from *S. cerevisiae*, which calculated their kinetic parameters toward various substrates [4]. Notably, Bat1 and Bat2 showed similarities in substrate preferences; specifically, the *k*_cat_/*K*_m_ value toward KIV was approximately 6–8 fold higher than the value toward valine. Indeed, both BCATs confer similar reaction preferences, distinctly favoring the biosynthesis of BCAAs over their degradation.

### 3.4 Media optimization for isobutanol production in IbOH-1*bat1*δ

Following the acquisition of IbOH-1*bat1∆* from the preceding section, we validated and assessed the isobutanol production in this *BAT1* knockout strain. Overall, IbOH-1*bat1∆* demonstrated a significantly greater elevation in isobutanol levels compared to the sub-quantified isobutanol content observed in IbOH-1 (Fig. 4). Since Bat1 drives bi-directional transamination reactions between the key intermediate KIV and amino acid valine (Val) [32]. Furthermore, the recent work elucidated that Bat1 of the yeast *S. cerevisiae* favors the synthesis of Val from KIV rather than the degradation of Val into KIV [4]. Thus, the inactivation or disappearance of Bat1 results in an accumulation of KIV, which subsequently advances to isobutanol synthesis through the KIVC pathway (including the transport of KIV across mitochondria and its conversion via the Ehrlich pathway) (see also, Fig. 1) [3,10]. We propose that the isobutanol overproduction mechanism in IbOH-1*bat1*Δ resembles that of other strains from prior studies, wherein the KIVC pathway facilitates the transport of accumulated KIV from the mitochondria to the cytosol, where it is subsequently converted to isobutanol by KDC and ADH enzymes [3,10].

**Fig. 5:**
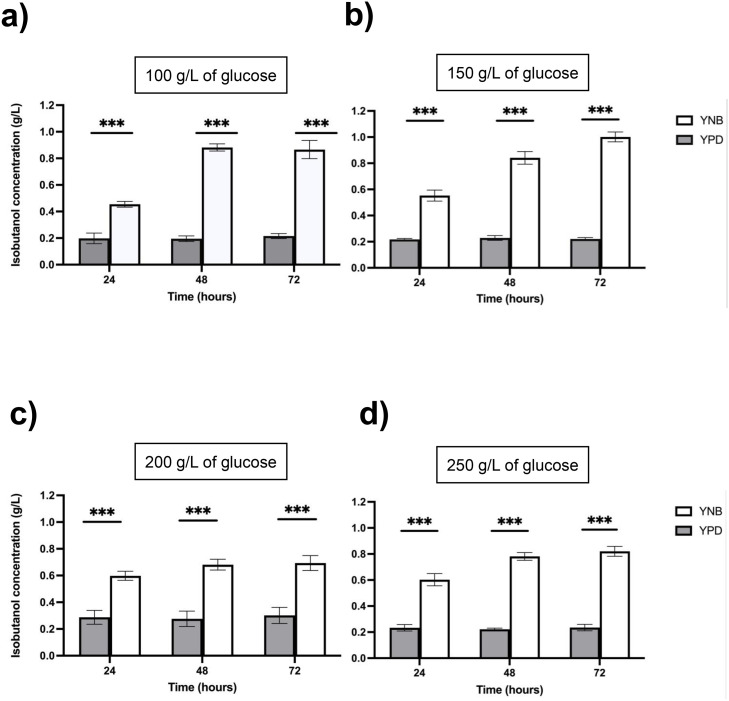
Isobutanol concentration (g/L) of IbOH-1*bat1∆* in YNB and YPD medium containing different concentrations of glucose. Statistically significant differences between cultivation in YNB and YPD were verified by Student’s *t*-test (***P < 0.001).

Nutritional conditions in the fermentation medium, particularly the presence or absence of the amino acid Val, significantly influenced the efficiency of isobutanol production [3]. Accordingly, we investigated isobutanol production in both nutrient-rich media YPD medium and minimal YNB medium devoid of valine. The isobutanol yield from IbOH-1*bat1∆* was 0.215 ± 0.04 g/L in YPD medium with 100 g/L of glucose after 72 h of culture (Fig. 5a). In contrast, fermentation using YNB medium supplemented with 100 g/L of glucose resulted in an isobutanol concentration of 0.866 ± 0.042 g/L by the IbOH-1*bat1*Δ cells after 72 hours of cultivation (Fig. 5a). The trend of significantly increased isobutanol titer when utilizing YNB medium as fermentation medium, compared to the values obtained from YPD medium, was evaluated under all fermentation conditions (Fig. 5a to 5d). The results showed that the nutritional content of isobutanol fermentation media, particularly the plentiful amino acid Val, is a significant determinant in the overproduction of isobutanol, as described in the previous study [3]. This leads from the regulation of valine amino acid biosynthesis; Val feedback inhibits the rate-limiting enzyme for Val biosynthesis: the regulatory subunit of acetohydroxy acid synthase [AHAS (Ilv6 encoded by the *ILV6* gene)] (Fig. 1) [[37], [38], [39]]. In turn, the amount of the crucial intermediate for isobutanol synthesis (KIV) is further restricted, resulting in a decrease in Val and isobutanol concentration [3,26]. A recent study indicated that some amino acid alterations in Ilv6 can desensitize the feedback inhibition of Val, allowing cells with these Ilv6 variants to restore Val levels [36]. Yeast cells included Val-insensitive Ilv6 mutants also capable of reinstating isobutanol productivity [3,26]. However, the incorporation of the Ilv6 variation necessitates additional engineering of yeast cells. Accordingly, Hammer and Avalos (2017) suggested a simplified method including fermentation media devoid of Val [3].

**Fig. 6:**
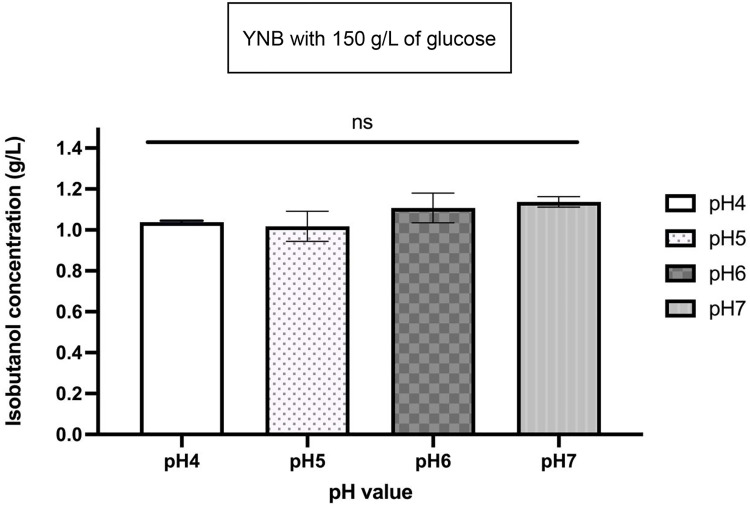
Isobutanol concentration (g/L) of IbOH-1*bat1∆* across different pH conditions. The statistically non-significant differences among different pH levels were verified by two-way ANOVA followed by Tukey’s multiple comparisons test (^ns^indicated non-significant differences).

To enhance isobutanol production, we further optimized the glucose concentration in both YPD and YNB media and performed fermentation using IbOH-1*bat1*Δ (Fig. 5). Consequently, increasing the glucose concentration in YNB medium from 100 g/L to 150 g/L boosted the isobutanol titer from 0.866 ± 0.042 g/L to 1.001 ± 0.038 g/L following 72 h of cultivation (Fig. 5a and b). Nevertheless, increasing glucose concentration in culture media to 200 and 250 g/L negatively affected the isobutanol titer (Fig. 5c and d). The isobutanol concentration decreased to 0.693 ± 0.056 g/L and 0.820 ± 0.038 g/L in YNB with 200 and 250 g/L of glucose, respectively, throughout a 72-hour cultivation time. pH was an auxiliary variable in the optimization of isobutanol production [27]. Consequently, we investigated the impact of pH fluctuations on isobutanol synthesis, with the fermentation media pH adjusted between 4.0 and 7.0 (Fig. 6). The results demonstrated that the isobutanol production from IbOH-1*bat1*Δ in YNB medium containing 150 g/L glucose showed no significant variation across the pH range of 4.0 to 7.0, indicating that this strain of yeast is naturally tolerant of pH. This finding is in accordance with a prior study by Liu et al. (2015), which showed that an industrial strain of *S. cerevisiae* could maintain comparable amounts of ethanol production in acidic environments (pH 2.5–4.5) [40]. The industrially isolated ethanol-producing yeast isolate G2–3–2 (D3C strain), from which IbOH-1*bat1*∆ developed, is expected to acquire the pH-insensitive bio-alcohol production [22]. To elucidate this pH-tolerant mechanism from IbOH-1*bat1*∆, further investigation is required.

**Fig. 7:**
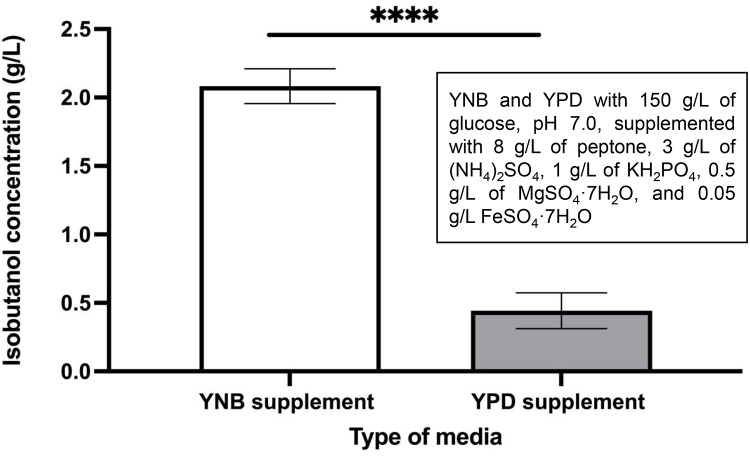
Isobutanol concentration (g/L) of IbOH-1*bat1∆* in nutrient supplemented YNB and YPD. Statistically significant differences between cultivation in modified YPD and YNB were verified by Student’s *t*-test (***P < 0.001).

A recent study by Ramli et al. (2021) employed response surface methodology (RSM) to optimize the fermentation medium for isobutanol production. RSM is a statistical approach used to evaluate the interactive effects of multiple medium components and to determine their optimal concentrations for maximizing product yield. In their study, seven factors glucose, (NH_4_)_2_SO₄, peptone, yeast extract, KH_2_PO_4_, MgSO_4_·7H_2_O, and FeSO_4_·7H_2_O were examined. The model and experimental data indicated that addition of 8 g/L of peptone, 3 g/L of (NH_4_)_2_SO_4_, 1 g/L of KH_2_PO_4_, 0.5 g/L of MgSO_4_·7H_2_O, and 0.05 g/L of FeSO_4_·7H_2_O markedly improves isobutanol production from *S. cerevisiae* [27]. In this experiment, YNB and YPD media containing 150 g/L glucose and adjusted to pH 7.0 were further enriched with selected minerals and trace elements. Consistent with the prior findings, the isobutanol concentration from IbOH-1*bat1*Δ demonstrated a markedly different value in YNB compared to YPD media (Fig. 7). Interestingly, the addition of 8 g/L of peptone, 3 g/L of (NH_4_)_2_SO_4_, 1 g/L of KH_2_PO_4_, 0.5 g/L of MgSO_4_·7H_2_O, and 0.05 g/L of FeSO_4_·7H_2_O greatly boosted isobutanol production from IbOH-1*bat1*∆ cells to 2.016 ± 0.127 g/L in YNB medium containing 150 g/L glucose at pH 7.0.

Regarding optimization of the fermentation media, the previous study reported that further supplementaion with a comprehensive trace elements, including MgSO_4_ at 3 g/L, ethylenediaminetetraacetic acid (EDTA) at 15 g/L, ZnSO_4_ at 5.75 g/L, MnCl_2_ at 0.32 g/L, CuSO_4_ at 0.5 g/L, CoCl_2_ at 0.47 g/L, Na_2_MoO_4_ at 0.48 g/L, CaCl_2_ at 2 g/L, and FeSO_4_ at 2.8 g/L, along with a vitamins into the fermentation media showed positive effect on isobutanol productivity [41]. Concerning the economic viability of the process, low-cost substrates could be employed as primary carbon sources. Substrates include sugarcane molasses, an abundant by-product from the sugar industry, utilized for its high fermentable sugar content, while lignocellulosic biomass was integrated as a sustainable feedstock to mitigate potential conflicts between the fuel and food industries [12].

### 3.5 Preliminary evaluation of isobutanol production performance in 5-L bioreactor

**Fig. 8:**
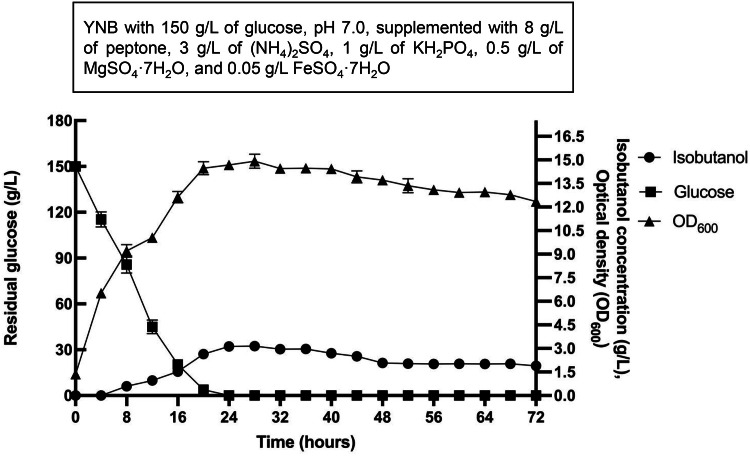
Fermentation profiles of the IbOH-1*bat1*∆ strain in a 5-L bioreactor with a 3-L working volume, showing isobutanol concentration (g/L), residual glucose (g/L), and optical density (OD_600_).

To evaluate the fermentation performance at a larger scale, a preliminary fermentation was conducted in a 5-L bioreactor. The IbOH-1*bat1*Δ strain was cultivated in nutrient-supplemented YNB under optimal conditions, and key parameters, including isobutanol concentration, residual glucose, and optical density at 600 nm (OD_600_), were monitored throughout the fermentation process (Fig. 8).

Initially (0 h), the glucose concentration and OD_600_ were 150.015 ± 0.004 g/L and 1.34 ± 0.126, respectively. Isobutanol production was first detected at 8 h, at which point glucose had decreased to 75.35 ± 7.574 g/L, while the OD_600_ increased to 9.34 ± 0.410 and isobutanol reached 0.80 ± 0.063 g/L. After 24 h, glucose was completely consumed (0 g/L), with the OD_600_ increasing to 14.64 ± 0.223. The maximum isobutanol concentration was observed at this time, reaching 3.12 ± 0.068 g/L. At the end of fermentation (72 h), the isobutanol concentration and OD_600_ had slightly decreased to 1.870 ± 0.103 g/L and 12.70 ± 0.213, respectively.

The results indicated that the high cell density inoculation strategy effectively shortened the lag phase and allowed the culture to reach its biosynthetic potential quickly, which is similar to a previous study [42]. However, following the complete depletion of glucose at 24 h, the isobutanol concentration began to decline, suggesting a limitation in substrate availability during the later stages. This observation aligns with previous studies, which reported a decrease in isobutanol yield upon glucose exhaustion [43,44]. Under bioreactor conditions, this decline is primarily driven by the gas stripping effect; continuous aeration and agitation cause the volatile isobutanol to be removed from the liquid phase once the production rate can no longer offset the evaporation rate [45].

Given that these results represent a preliminary scale-up to a bioreactor, several avenues for bioprocess optimization will be explored in future studies to enhance the final isobutanol titer and productivity. First, a fed-batch fermentation strategy will be implemented to overcome the substrate limitation observed at 24 h. Maintaining a stable glucose concentration throughout the process prevents premature substrate depletion and sustains metabolic activity, thereby prolonging the active production phase [41]. Lane et al. (2019) also demonstrated that switching the fermentation mode from batch to fed-batch could enhance isobutanol production from 2.72 ± 0.10 g/L to 3.10 ± 0.18 g/L [42]. Additionally, the optimization of the inoculum ratio is proposed as a key strategy. While the current high cell density approach used in this study successfully shortened the lag phase, the rapid glucose depletion by 24 h suggests that the biomass concentration exceeded the batch capacity of the system [42]. Therefore, fine-tuning the inoculum size should be evaluated to balance the substrate consumption rate with the timeframe required for isobutanol accumulation [41]. Lastly, optimizing the balance between airflow rate (0.5–1.5 vvm) and agitation speed (50–250 rpm) will ensure that dissolved oxygen (DO) levels remain sufficient to support the metabolic flux toward isobutanol while maximizing overall process efficiency [28,41,46].

### 3.6 Preliminary genomic characterization of isobutanol tolerance mechanism

**Fig. 9:**
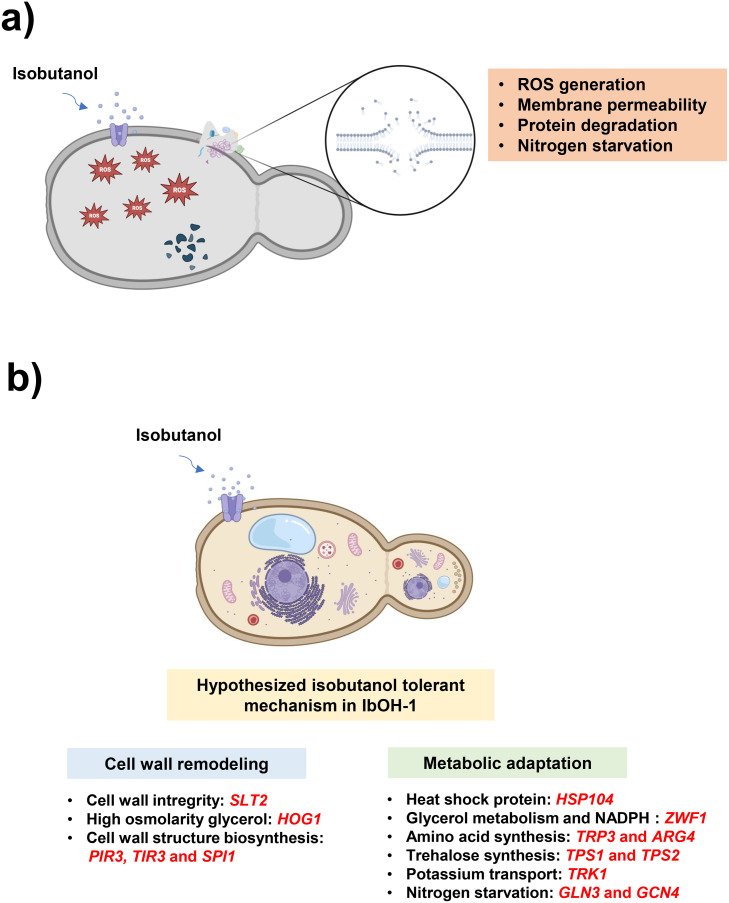
Mutations in pathways associated with isobutanol stress tolerance in *Saccharomyces cerevisiae* IbOH-1. (a) Mechanisms of isobutanol toxicity to yeast cells, and (b) the hypothesized isobutanol tolerance mechanism in the IbOH-1 strain. Genes in red indicated the mutations identified in IbOH-1 compared to the parental strain G2–3–2.

Isobutanol influences the cell membrane and cell wall by enhancing membrane permeability and causing damage [47] and inducing nitrogen starvation [19], leading to protein misfolding and degradation [48]; correspondingly, cell death [49]. and lowering the productivity of isobutanol [19]. Previous studies discovered that mutations in genes associated with TATA-binding protein, serine‑rich protein, pentose phosphate pathway, tryptophan synthesis, nitrogen starvation response, potassium transport, high osmolarity glycerol response, cell wall integrity, cell wall biogenesis, arginine synthesis, and heat shock protein pathways in isobutanol-tolerant yeast facilitated the cells' ability to withstand isobutanol toxicity (Fig. 9) [19,[50], [51], [52], [53], [54], [55], [56], [57]]. Hence, we focused on genes involved in the isobutanol tolerance mechanism in IbOH-1, which can tolerate up to 21 g/L of isobutanol (Fig. 2) by performing a comparative genomic DNA analysis against the parental strain G2–3–2.

The whole genome sequencing results of IbOH-1 identified a comprehensive spectrum of genomic variations, including nucleotide insertions, deletions, and single nucleotide polymorphisms (SNPs). These mutations were distributed throughout the genome, affecting both open reading frames (ORFs) and non-coding regulatory regions (i.e., upstream and downstream sequences). Specifically, IbOH-1 possessed numerous mutations in the gene associated with isobutanol tolerance, encompassing signaling, cell wall, cell membrane, and amino acid pathways (Fig. 9). Mutations were identified in several important pathways associated with isobutanol tolerance. The entities encompassed the TATA-binding protein (*SPT15*, with 1 synonymous mutation), serine‑rich proteins (*SRP40*, featuring 2 conservative in-frame deletions), the pentose phosphate pathway (*GND1, ZWF1, TKL1, SOL3*, exhibiting 10 synonymous and 2 missense mutations), nitrogen starvation response (*GLN3, GCN4*, comprising 4 synonymous and 4 missense mutations), potassium transport (*TRK1*, incorporating 6 synonymous and 11 missense mutations), and heat shock proteins (*HSP60, HSP104*, including 2 synonymous and 1 missense mutation). A significant number of mutations was noted in pathways related to cell wall and cell membrane integrity, particularly the high-osmolarity glycerol (HOG) pathway (*SHO1, SSK1, SKN7, SSK2, SSK22, PBS2, HOG1, PTP2, PTP3, SLT2*), which exhibited 44 synonymous, 13 missense, 2 conservative in-frame, and 1 disruptive in-frame deletion. Moreover, genes associated with cell wall formation (*SLT2, HSP150, CHS1, ACF2, PIR3, SED1, SPI1, TIR3, TIP1, KRE5*) exhibited 56 synonymous mutations, 13 missense mutations, 1 disruptive in-frame mutation, and 1 frameshift mutation. The cell wall integrity (CWI) pathways (*WSC2, WSC3, PKC1, SLT2, RLM1, SWI4*) comprised 13 synonymous mutations, 7 missense mutations, 1 conservative in-frame mutation, 2 disruptive in-frame mutations, and 3 upstream mutations. Further mutations were detected in genes related to amino acid biosynthesis, specifically in the tryptophan pathway (*PRS3, ARO1, ARO2, TRP4, TRP1, TRP3, TRP5*, comprising 25 synonymous and 5 missense mutations) and the arginine synthesis pathway (*ARG5, ARG6*, including 3 synonymous and 1 missense mutation), which are associated with redox balance and stress resilience. The data indicated that IbOH-1 harbors mutations in various stress-related pathways, potentially enhancing its isobutanol tolerance (Table 2 and **Supplementary table S3**).

**Table 2:** The pathways associated with isobutanol toxicity tolerance and the mutations identified in the IbOH-1 mutant within genes related with those pathways.

**Pathway**	**Function**	**Type of mutations**	**Number of mutations**
TATA Binding protein	RNA Pol II transcription factor D (TFIID) and expression gene involved in isobutanol tolerance (Zhang et al., 2021)	Synonymous	1
Serine rich protein	Pre-ribosome assembly or transport related to isobutanol tolerance and production (Zhang et al., 2024)	Conservative inframe deletion	2
Pentose phosphate pathway	Maintaining cellular redox homeostasis by reducing NADP^+^ to NADPH in stress responses (Kuroda et al., 2019)	Synonymous	10
		Missense	2
Tryptophan synthesis	Cellular redox balance and anti-oxidative ability (Liu et al., 2021)	Synonymous	25
		Missense	5
Nitrogen starvation	Nitrogen starvation response inhibits glycolysis and HXT expression (Kuroda et al., 2019)	Synonymous	4
		Missense	4
Transportation of potassium	Ion homeostasis related to alcohol sensitivity and tolerance (Lam et al., 2014)	Synonymous	6
		Missense	11
HOG pathway	Expression of genes involved in stress response including encode enzyme for glycerol synthesis to help balance of stress (Ribeiro et al., 2022)	Synonymous	44
		Missense	13
		Conservative inframe deletion	2
		Disruptive inframe deletion	1
Cell wall integrity	Maintaining the structural integrity of cell wall and responding to stress (Ribeiro et al., 2022)	Synonymous	13
		Missense	7
		Conservative inframe deletion	1
		Disruptive inframe deletion	2
		Upstream	3
Cell wall biogenesis gene	Cell wall polysaccharide synthases and enzyme involved in cell remodeling (Ribeiro et al., 2022)	Synonymous	56
		Missense	13
		Disruptive inframe deletion	1
		Frameshift	1
Arginine synthesis	Enhancing cell wall and membrane integrity, stabilizing organelle and reducing Reactive Oxygen Species (ROS) production (Cheng et al., 2016)	Synonymous	3
		Missense	1
Heat shock protein	Stabilize cell membranes and facilitate protein folding and assembly (Crook et al., 2016)	Synonymous	2
		Missense	1

The attachment of isobutanol to the cell membrane of *S. cerevisiae* activates stress responses and transmits signals that induce nitrogen deprivation [19]; simultaneously, convey signals to the receptors of cell wall integrity (CWI), genes involved in cell wall biogenesis, and the high osmolarity glycerol (HOG) pathway to maintain cell wall integrity and promote cell wall synthesis [54]. Concurrently, the increase of heat shock protein [55]. and stimulate the expression of tryptophan, arginine, and serine‑rich proteins to achieve redox equilibrium [50,53,56], accumulation of glycerol [58], inducing cellular mechanisms including transportation of potassium [52]. and pentose phosphate pathway [59]. were observed. The processes collectively aid yeast cells in tolerating the toxicity of isobutanol. This study identified various mutations in IbOH-1 related to isobutanol tolerance pathways, including tryptophan and arginine synthesis, cell membrane pathways such as cell wall integrity, the high-osmolarity glycerol (HOG) pathway, glycerol accumulation, potassium transport, the pentose phosphate pathway, and TATA-binding protein.

*HOG1* and *SLT2*, key genes in the HOG and CWI pathways respectively, were found to harbor novel mutations in the IbOH-1 strain. Specifically, *HOG1* exhibited a conservative in-frame deletion at nucleotide (nt) 168. In parallel, its negative regulators, *PTP2* and *PTP3*, carried missense mutations at positions 553 and 675, respectively, along with a distinct in-frame deletion at nt 589. Under physiological conditions, Ptp2 and Ptp3 dephosphorylate and terminate Hog1 signaling [60]. The simultaneous structural alteration of these interconnected components strongly suggests a complex pathway-level rewiring. Since isobutanol acts as a severe chaotropic agent that fluidizes the plasma membrane—a stress fundamentally similar to osmotic shock [61].—we hypothesize that these combined mutations might disrupt the negative feedback loop. This could effectively shift the HOG pathway into a more constitutively active state. Consequently, the altered signaling dynamic would presumably enhance the nuclear translocation of Hog1 under alcohol-induced stress, leading to the upregulated expression of glycerol synthesis genes (e.g., *GPD1, GPP2*) and promoting intracellular glycerol accumulation as a protective response [62].

Similarly, the IbOH-1 strain exhibits strategic structural variations within the Cell Wall Integrity (CWI) pathway. As a key MAP kinase in the CWI pathway, Slt2 regulates transcription factors such as Rlm1 to drive cell wall remodeling and maintain structural integrity under stress [58]. In the IbOH-1 strain, a disruptive in-frame deletion was identified in *SLT2* (at nt 1123), accompanied by a comparable mutation in its primary downstream transcription factor, *RLM1* (at nt 1706). While the canonical CWI cascade is vital for repairing surface damage, continuous hyperactivation under constant isobutanol stress could trap the cell in a futile, energy-draining cycle. Therefore, we hypothesize that these mutations might serve as an attenuation mechanism, allowing the cell to bypass unnecessary metabolic costs while maintaining a specialized level of tolerance. Ultimately, these structural alterations may establish a pre-activated-like state, enhancing glycerol accumulation, cell wall remodeling, and other stress responses, thereby fortifying IbOH-1 against isobutanol-induced stress [[62], [63], [64]].

The previous work identified that mutations in the promoter, namely a deletion at −162 bp and an insertion at −97 bp, of the *SPT15* gene in *S. cerevisiae* augment isobutanol tolerance by modifying the transcription of *SPT15* [57]. The *SPT15* gene encodes the TATA-binding protein (TBP), an essential regulator of gene expression. Our study discovered a mutation in the *SPT15* promoter, albeit at a different locus. These mutations may similarly enhance *SPT15* expression, thereby facilitating enhanced isobutanol tolerance. Furthermore, whole genome sequencing revealed nucleotide changes in the promoter region of *GLN3* including upstream regulatory alterations an insertion at nt −171 and a deletion at nt −60 (Table 2 and **Supplementary table S3**), a transcriptional activator implicated in nitrogen catabolite repression (NCR) [65], as well as a point mutation at position 706 (G > T) leading to a serine‑to-isoleucine amino acid substitution at residue 236 (Ser236Ile). These mutations may impair *GLN3* transcription and hinder post-translational modifications of the Gln3 protein, such as phosphorylation, or perhaps create conformational alterations that influence its function. Such modifications are anticipated to compromise Gln3 functionality, possibly replicating the phenotypic effects observed in *GLN3* deletion mutants. The deletion of *GLN3* greatly enhances yeast tolerance to branched-chain alcohols and improves isobutanol synthesis [19].

Beyond intracellular signaling, the genomic DNA sequencing data of IbOH-1 also revealed mutations in genes associated with ion homeostasis and membrane stabilization. The *TRK1* gene, encoding the primary high-affinity potassium transporter, accumulated an unusually high density of missense mutations (at 11 positions). Since isobutanol permeabilizes the membrane and causes intracellular ion leakage, robust potassium uptake is critical for restoring the plasma membrane potential [66]. These clustered mutations in *TRK1* may alter potassium affinity to rapidly compensate for solvent-induced leakage. Additionally, several missense mutations were identified in genes involved in the tryptophan biosynthesis pathway (e.g., *ARO1, TRP1, TRP3, TRP4*). Tryptophan, a highly hydrophobic amino acid, is pivotal for membrane stabilization and acts as a potent scavenger of reactive oxygen species (ROS) during solvent stress [53,67]. Mutations in these key genes may alleviate feedback inhibition, driving metabolic flux toward tryptophan overproduction to fortify the membrane interface.

In conclusion, this preliminary genomic characterization of the IbOH-1 strain indicates that the synergistic accumulation of mutations across the HOG, CWI, and NCR pathways, as well as in genes related to ion homeostasis and amino acid biosynthesis, could confer robust isobutanol tolerance to the IbOH-1 strain. However, it is important to note that the proposed mechanisms remain hypothetical, as they are derived from genomic analysis alone. Therefore, future studies will unveil the isobutanol tolerance mechanisms in IbOH-1 using comprehensive transcriptomic, proteomic, and metabolomic profiling, together with targeted multi-gene complementation assays, to elucidate the detailed physiological responses to isobutanol toxicity [68].

### 3.7 Comparison of isobutanol production performance from IbOH-1*bat1*δ

To evaluate the improvement in isobutanol productivity, the results obtained from our engineered strain were compared with those reported in previous studies on *S. cerevisiae* (Table 3) [[69], [70], [71]]. Su et al. (2021) modified the mutant derived from the laboratory strain W303–1A, which exhibits tolerance to 20 g/L of isobutanol, and further engineered it to augment isobutanol production by overexpressing gene associated with isobutanol biosynthesis, including *ILV2, ILV3*, and *ARO10*. As a result, the modified strain produced 0.404 g/L of isobutanol after 32 h of incubation [16]. Hammer and Avalos (2017) endeavored to enhance isobutanol production using the laboratory yeast strain (CEN.PK2–1C) through a metabolic engineering strategy. The maximum isobutanol concentration achieved was 1.25 g/L through the deletion of *BAT1*, along with the overexpression of *ILV2, ILV3*, and *ILV5*, as well as the mitochondrial compartmentalization of downstream biosynthetic enzymes *ARO10* and LiAdh*ARE1* [3]. Hoffman et al. (2021) constructed the engineered laboratory strain YEZ546–2 with deletions of genes related to glucose fermentation and overexpressing *ILV2*, was capable of cellulose utilization and achieved 0.364 g/L isobutanol under anaerobic conditions [72].

**Table 3:** Comparison of isobutanol concentration between the metabolic engineering laboratory strain of *S. cerevisiae* from previous studies and the newly developed IbOH-1*bat1*∆.

***S. cerevisiae* strain**	**Culture media**	**Fermentation Scale**	**Metabolic engineering**	**Isobutanol titer (g/L)**	**Isobutanol yield (mg isobutanol/g substrate)**	**Reference**
D-I253 (lab strain)	YPD medium contain 40 g/L glucose	Shake flask 250 mL (working volume 50 mL)	D452–2/ pRS425 GPD/p426*ILV253*	0.151	3.78 mg isobutanol/g glucose	(Lee et al., 2012)
HZAL-7023 22-*ZWF1* (lab strain)	SC media supplemented with appropriate amino acids and 40 g/L glucose	Shake flask 250 mL (working volume 100 mL)	W303–1A *PGK1p-BAT2 pdc6*Δ*∷R* YEplac181-*PGK1p-ILV2* YEplac195-*PGK1p-ILV3* YCplac22-*ZWF1*	0.283	11 mg isobutanol/g glucose	(Feng et al., 2017)
SHy48 (lab strain)	SC medium lacking uracil and valine with 100 g/L glucose	24-well plates(working volume 1 mL)	CEN.PK2–1C *bat1Δ::hygMX* (*ILV2, ILV3, ILV5,* Cox4*-ARO10,* Cox4-*LlAdhA^RE1^*)	1.245	12.45 mg isobutanol/g glucose	(Hammer & Avalos, 2017)
BY4743 *gln3Δ/gln3Δ* (pJA184) (lab strain)	SC-Ura medium with 150 g/L glucose	Falcon tube 14 mL (working volume 5 mL)	BY4743 *gln3*Δ*::kanMX4/gln3Δ::kanMX4* pJA184 (*ILV2, ILV3, ILV5, LlKivD, ADH7*)	0.809	-	(Kuroda et al., 2019)
JWY23 (lab strain)	SC medium supplemented with amino acids lacking valine and 40 g/L glucose	Shake flask 100 mL (working volume 50 mL)	CEN.PK113–7D *Δilv2;Δbdh1;Δbdh2;Δleu4;Δleu9;Δecm31;Δilv1;Δadh1;Δgpd1;Δgpd2; Δald6;*2µ-plasmid IsoV100 (Ilv2Δ54; Ilv5Δ48; Ilv3Δ19)	2.090	59.55 mg/g glucose	(Wess et al., 2019)
JHY43D24 (lab strain)	SC medium supplemented with 20 μM CuSO_4_ and 20 g/L glucose	Conical tube 50 mL (working volume 6.5 mL)	JHY43D2 with random multiple integration of P*_TDH3_*-(K)*ILV5ΔN48-*T*_CYC1_*, P*_TDH3_-*(K)*ILV3ΔN19-*T*_CYC1_* at NTS sites	0.263	13.16 mg isobutanol/g glucose	(Park & Hahn, 2019)
PWY2353 (lab strain)	SX selective yeast medium containing xylose 20 g/L	Shake flask 250 mL (working volume 50 mL)	PWY2343 *PHO13*Δ	0.047	7.0 mg isobutanol/g D-xylose	(Promdonkoy et al., 2019)
SR8-Iso (lab strain)	Nutrient rich Verduyn (NRV) medium supplement with trace element,vitamins and contain xylose 80 g/L	Bioreactor (working volume 1 L)	SR7e3 *ald6*::*AUR1*-C pAUR_d_ALD6 expressing (*S. stipitis XYL1, XYL2*, and *XYL3*)) harboring pJA180	2.6	32.5 mg isobutanol/g glucose	(Lane et al., 2019)
PWY2353ev34b (lab strain)	SX-URA containing xylose 25 g/L	Shake flask 250 mL (working volume 50 mL)	PWY2353ev34 *ARS416:: P TDH3 –LlkivDmt-T2A-ScADH7mt; ARS1014:: P TEF1-ScIlv2-T2A-ScIlv5-T2A-ScIlv3*	0.092	3.72 mg isobutanol/g D-xylose	(Promdonkoy et al., 2020)
YEZ546–2 (lab strain)	SC-Ura supplemented with 2% cellulose sodium acetate buffer (270 mM) and Acellerase 1500 cellulase enzymes	Shake flask 125 mL (working volume 25 mL)	YEZ532 YARCdelta5::(PC120_*PDC1*_TACT1, PGAL1-M_*ILV2*_TADH1) minus *pJLA121-PDC10202*+ EZ-L390	0.364	-	(Hoffman et al., 2021)
EMS39–20 (lab strain)	YPD medium containing 40 g/L glucose	Shake flask 250 mL (working volume 100 mL)	EMS39 YEplac195-PGK1p-ILV2 YEplac112-PGK1p-ILV3 YEplac181-TDH3p-cox4-*ARO10*	0.404	10.41 mg isobutanol/g glucose	(Su et al., 2021)
*Engi3* (lab strain)	YPD medium containing 60 g/L glucose	Shake flask 250 mL (working volume 100 mL)	W303‐1A δ*5′*‐*HIS3*‐*PGK1p*‐*ILV3*‐δ*3′*, YEplac195‐*PGK1p*‐*ILV2*, YEplac181‐*TDH3p*‐*cox4*‐*ARO10*‐*GFP*‐*CYC1*, YCplac22‐*SPT15*	0.556	8.49 mg isobutanol/g glucose	(Zhang et al., 2021)
303V2V3A10–22-*srp40* (lab strain)	YPD medium containing 60 g/L glucose	Shake flask 250 mL (working volume 100 mL)	W303–1A *δ::HIS3*-*PGK1p*-*ILV3* YEplac195-*PGK1p*-*ILV2* YEplac181-*TDH3p*-*COX4*-*ARO10*-*GFP*-*CYC1* YCplac22-*srp40*	0.385	6.61 mg isobutanol/g glucose	(Zhang et al., 2024)
IbOH-1*bat1∆* (derived from the newly isolated G2–3–2)	YNB medium supplement with minerals and containing 150 g/L glucose	Shake flask 250 mL(working volume 50 mL)	IbOH-1*bat1∆*	2.016	15.11 mg isobutanol /g glucose	This study
IbOH-1*bat1∆* (derived from the newly isolated G2–3–2)	YNB medium supplement with minerals and containing 150 g/L glucose	5 L bioreactor (working volume 3 L)	IbOH-1*bat1∆*	3.12	*20.8mg* isobutanol*/g glucose*	This study

In contrast to previous studies that utilized laboratory strains with extensive genetic and metabolic engineering, this study developed IbOH-1 demonstrated tolerance to 21 g/L of isobutanol and IbOH-1*bat1*∆ cells achieved the highest isobutanol production at the shake-flask scale, yielding a titer of 2.016 g/L, with an isobutanol yield of 15.11 mg/g glucose (3.69% of the theoretical maximum yield). The isobutanol titer obtained in this study was in a similar range to that reported by Wess et al. (2019). The laboratory strain CEN.PK113–7D was modified through overexpression of Ilv2, Ilv5, and Ilv3, disruption of endogenous *ILV2* gene, and deletion of competing pathways (*BDH1, BDH2, LEU4, LEU9, ECM31, ILV1*), glycerol synthesis genes (*GPD1, GPD2*), and the by-product-associated gene *ALD6*. The resulting strain achieved an isobutanol titer of 2.09 g/L [73].

When considering performance in bioreactor systems, our engineered IbOH-1*bat1*∆ strain demonstrated competitive results despite having a minimal genetic modification background. For instance, Lane et al. (2019) achieved an isobutanol titer of 2.6 g/L using the SR8-Iso strain, which required extensive genetic engineering, including addition of *XYL1, XYL2, XYL3* genes, a *PHO13* mutation, an *ALD6* deletion, and the overexpression of the *ILV2, ILV3*, and *ILV5* genes, combined with a fed-batch strategy in working volume 1-L bioreactor [41]. In comparison, our study achieved a comparable isobutanol titer of 3.12 g/L in a 5-L bioreactor with a 3-L working volume, with an isobutanol yield of 20.8 mg/g glucose (5.06% of the theoretical maximum yield) using batch operation strategy. This performance represents a 1.55-fold increase compared to the 2.016 ± 0.127 g/L obtained at the initial shake-flask scale. The transition from shake flasks to the 5-L bioreactor markedly enhanced the production performance, which is likely due to the superior oxygen transfer efficiency and enhanced mass transfer within the bioreactor system [28], thereby facilitating the carbon flux towards isobutanol production pathway [74].

Thus, our findings highlight the significance of strain selection and development for industrial isobutanol production, demonstrating that minimal genetic modification, specifically *BAT1* gene knockout, combined with the supplementation of essential trace elements, can highly enhance isobutanol yield. Notably, the performance was maintained—and even enhanced—when scaled up to a 5-L bioreactor. In fact, several extensively genetically engineered strains suffer from the harsh bioreactor conditions, particularly the shear forces generated by intensive aeration and agitation systems [75]. This confirms the robustness and industrial potential of the IbOH-1*bat1*∆ strain developed in this study.

## 4 Conclusion

The present study successfully developed an industrially applicable isobutanol-producing strain from the newly isolated osmotolerant ethanol-producing *S. cerevisiae* D3C from the Thai sugar industry: Thai Multi-Sugar Industry, utilizing CRISPR-Cas9 mediated gene knockout of the rate-limiting enzymes to eliminate the need for cloning vectors and antibiotic markers, thereby reducing maintenance costs in industrial processing. The resultant strain was able to highly increase isobutanol productivity. This foundational work could be expanded by optimizing and scaling up fermentation conditions and substrate selection to boost isobutanol productivity, thereby meeting industrial demand at an acceptable production cost. Moreover, a comprehensive understanding of the isobutanol tolerance mechanism merits further exploration, which could yield new discoveries.

Supplementary data containing all essential genetic information—specifically, the nucleotide sequences of the *BAT1* gene, single guide RNA, and the identified tolerance-related nucleotide substitutions—is available online.

## Declaration of generative AI and AI-assisted technologies in the manuscript preparation process

During the preparation of this work the author(s) used QuillBot and Google Gemini in order to proofread the text and improve readability. After using these tools, the author(s) reviewed and edited the content as needed and take(s) full responsibility for the content of the published article.

## CRediT authorship contribution statement

**Naphattarachon Thammapanyaphong:** Writing – review & editing, Writing – original draft, Validation, Methodology, Investigation, Formal analysis, Data curation. **Manutsanun Boonyanuwat:** Validation, Investigation, Data curation. **Apanee Luengnaruemitchai:** Writing – review & editing, Writing – original draft, Validation, Supervision, Methodology, Formal analysis, Funding acquisition, Conceptualization. **Jirasin Koonthongkaew:** Writing – review & editing, Writing – original draft, Validation, Supervision, Methodology, Funding acquisition, Formal analysis, Conceptualization.

## Declaration of competing interest

We declare no conflicts of interest.

## Data Availability

No data was used for the research described in the article.

## Appendix Supplementary materials

Image, application 1
