# Supplementary material for: Development of Saccharomyces cerevisiae isobutanol production strain from osmotolerant and ethanol-producing industrial isolated yeast
Source: Biotechnol Rep (Amst). 2026 Apr 22;50:e00959. doi: 10.1016/j.btre.2026.e00959 (PMC13137203; doi:10.1016/j.btre.2026.e00959)
Supplement: Supplementary file 1 [file mmc1.docx]

**Supplementary Material**

**Development of *Saccharomyces cerevisiae* Isobutanol Production Strain From Osmotolerant and Ethanol-producing Industrial Isolated Yeast**

Naphattarachon Thamapanyaphong^1^, Manutsanun Boonyanuwat^1^, Apanee Luengnaruemitchai^3,4,5^, Jirasin Koonthongkaew^1,2#^

^1^Department of Microbiology, Faculty of Sciences, Chulalongkorn University, Phayathai Rd., Pathumwan, Bangkok 10330, Thailand.

^2^Research Unit in Bioconversion/Bioseperation for Value-Added Chemical Production, Chulalongkorn University, Bangkok 10330, Thailand.

^3^The Petroleum and Petrochemical College, Chulalongkorn University, Bangkok 10330, Thailand.

^4^Center of Excellence on Catalysis for Bioenergy and Renewable Chemicals (CBRC), Chulalongkorn University, Bangkok 10330, Thailand.

#Corresponding author: Jirasin Koonthongkaew

E-mail: Jirasin.K@chula.ac.th, Tel: (+66)2-218-5085/ (+66)92-247-3816

Address: Department of Microbiology, Faculty of Science, Chulalongkorn University, 16th Floor Maha Vajirunhis Building, 254 Phayathai Road, Patumwan, Bangkok, 10330 Thailand.

**This file includes Figure S1 and Table S1-S3**

**Figure S1** *BAT1* gene sequence comparison between G-2-3-2 (wild type) and IbOH-1 (mutant)


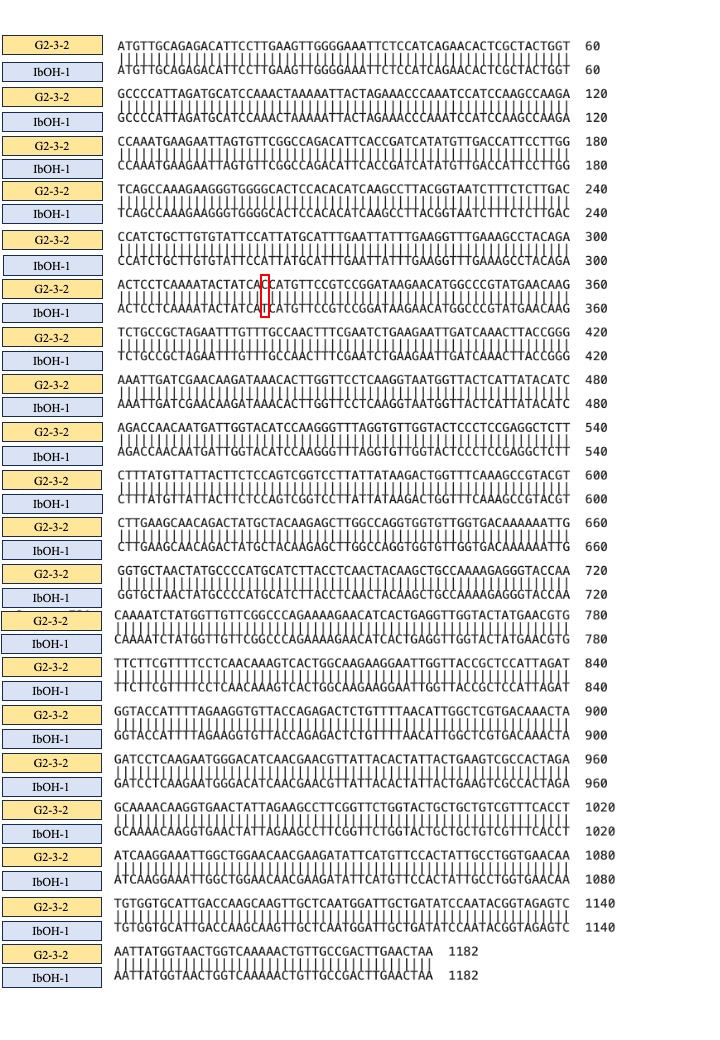


**Table S1** Single guide RNA sequence and PCR amplification primer in this study

| **sgRNA sequence** 5‘-TCAAGAGAAAGATTACCGTAAGG-3’ |
| --- |
| **PCR amplification primers** S035659-JJF (5‘-TTAACGCTGGATAAGTACCGC-3’)  S035659-JJR (5‘-CAACACCTAAACCCTTGGATG-3’) |

**Table S2** Nucleotide sequence of *BAT1* gene (from 5’ end to 3’ end) in IbOH-1*bat1*Δ (the bonded- and red-fonts represented start and stop codon, respectively)

| **Gene name** | **Sequence** |
| --- | --- |
| *BAT1* knockout in IbOH-1*bat1Δ* | **ATGTAA**TACCCATCTGCTTGTGTATTCCATTATGCATTTGAATTATTTGAAGGTTTGAAAGCCTACAGAACTCCTCAAAATACTATCACTATGTTCCGTCCGGATAAGAACATGGCCCGTATGAACAAGTCTGCCGCTAGAATTTGTTTGCCAACTTTCGAATCTGAAGAATTGATCAAACTTACCGGGAAATTGATCGAACAAGATAAACACTTGGTTCCTCAAGGTAATGGTTACTCATTATACATCAGACCAACAATGATTGGTACATCCAAGGGTTTAGGTGTTGGCACTCCCTCCGAGGCTCTTCTTTATGTTATTACTTCTCCAGTCGGTCCTTATTATAAGACTGGTTTCAAAGCCGTACGTCTTGAAGCAACAGACTATGCTACAAGAGCTTGGCCAGGTGGTGTTGGCGACAAAAAATTGGGTGCTAACTATGCCCCATGCATCTTACCTCAACTACAAGCTGCCAAAAGAGGGTACCAACAAAATCTATGGTTGTTCGGCCCAGAAAAGAACATCACTGAGGTTGGTACTATGAACGTGTTCTTCGTTTTCCTCAACAAAGTCACTGGCAAGAAGGAATTGGTTACCGCTCCATTAGATGGTACCATTTTAGAAGGTGTTACCAGAGACTCTGTTTTAACATTGGCTCGTGACAAACTAGATCCTCAAGAATGGGACATCAACGAGCGTTATTACACTATTACTGAAGTCGCCACTAGAGCAAAACAAGGTGAACTATTAGAAGCCTTCGGTTCTGGTACTGCTGCTGTCGTTTCACCTATCAAGGAAATTGGCTGGAACAACGAAGATATTCATGTTCCACTATTGCCTGGTGAACAATGTGGTGCATTGACCAAGCAAGTTGCTCAATGGATTGCTGATATCCAATACGGTAGAGTCAATTATGGTAACTGGTCAAAAACTGTTGCCGACTTGAACTAA |

**Table S3** Mutations in genes related to isobutanol toxicity tolerance

| **TATA Binding protein** | | | | | |
| --- | --- | --- | --- | --- | --- |
| **gene** | **position** | **Nucleotide original**  **(G2-3-2)** | **Nucleotide changes** **(IbOH-1)** | | **Type of mutations** |
| *SPT15* | -353 | A | G | | Substitutions |
|  | -291 | GAAAAAAAAAAAA | GAAAAAAAAAAA | | Deletion |
|  | -162 | GTTTTTTTTT | GTTTTTTTT | | Deletion |
|  | -97 | A | G | | Substitutions |
|  | 440 | G | A | | Synonymous |
| **Serine-rich protein** | | | | | |
| *SRP40* | 995 | AGTCACTGCTGTCACTGCT  GTCACTGCTGTCACTGCTG | AGTCACTGCT  GTCACTGCTG | | Conservative inframe deletion |
|  | 1116 | GGAGGAAGAGGAAGA | GGA | | Conservative inframe deletion |
| **Pentose phosphate pathway** | | | | | |
| *GND1* | 245 | T | A | | Synonymous |
| *ZWF1* | 1323 | T | C | | Synonymous |
|  | 1485 | G | A | | Synonymous |
|  | 1508 | G | A | | Missense |
| *TKL1* | 1065 | T | C | | Synonymous |
|  | 1221 | C | T | | Synonymous |
| *SOL3* | 147 | A | G | | Synonymous |
|  | 195 | G | A | | Synonymous |
|  | 234 | A | G | | Synonymous |
|  | 324 | G | A | | Synonymous |
|  | 364 | T | C | | Missense |
|  | 603 | G | A | | Synonymous |
| **Typtophan synthesis** | | | | | |
| *PRS3* | 264 | T | C | | Synonymous |
|  | 340 | C | T | | Synonymous |
|  | 417 | A | G | | Synonymous |
|  | 594 | G | A | | Synonymous |
|  | 792 | T | C | | Synonymous |
| *ARO1* | 11 | T | C | | Missense |
|  | 1897 | C | T | | Synonymous |
|  | 2547 | G | A | | Synonymous |
|  | 2649 | G | A | | Synonymous |
|  | 3252 | A | G | | Synonymous |
|  | 3366 | T | C | | Synonymous |
|  | 3435 | A | G | | Synonymous |
|  | 3846 | C | T | | Synonymous |
| *ARO2* | 504 | T | C | | Synonymous |
|  | 588 | C | G | | Synonymous |
|  | 627 | A | G | | Synonymous |
| *TRP4* | 339 | T | C | | Synonymous |
|  | 396 | A | G | | Synonymous |
|  | 456 | C | T | | Synonymous |
|  | 816 | T | C | | Synonymous |
|  | 880 | A | G | | Missense |
| *TRP1* | 314 | C | T | | Missense |
|  | 419 | A | G | | Synonymous |
|  | 453 | A | C | | Synonymous |
| *TRP3* | 112 | T | G | | Missense |
|  | 761 | G | A | | Missense |
| *TRP5* | 360 | A | G | | Synonymous |
|  | 492 | G | A | | Synonymous |
|  | 1122 | A | G | | Synonymous |
|  | 2034 | C | T | | Synonymous |
| **Nitrogen starvation** | | | | | |
| *GLN3* | -171 | ACA | ACAGGTTCCTGAAAGAAAGTGACATGGCA | | Insertion |
|  | -60 | AAGAGAGACGAGAGAGACGAGAGAGA | AAGAGAGACGAGAGAGA | | Deletion |
|  | 14 | C | T | | Synonymous |
|  | 641 | T | C | | Synonymous |
|  | 706 | G | T | | Missense |
|  | 2102 | A | G | | Synonymous |
| *GCN4* | 258 | T | G | | Missense |
|  | 351 | C | A | | Synonymous |
|  | 602 | T | C | | Missense |
|  | 802 | T | C | | Missense |
| **Transportation of potassium** | | | | | |
| *TRK1* | 101 | A | G | | Synonymous |
|  | 182 | G | A | | Synonymous |
|  | 278 | G | A | | Synonymous |
|  | 543 | A | C | | Missense |
|  | 691 | G | T | | Missense |
|  | 734 | T | C | | Synonymous |
|  | 853 | A | G | | Missense |
|  | 1183 | A | C | | Missense |
|  | 1329 | C | A | | Missense |
|  | 1614 | G | A | | Missense |
|  | 1940 | T | C | | Synonymous |
|  | 2067 | G | A | | Missense |
|  | 2094 | A | G | | Missense |
|  | 2797 | G | A | | Missense |
|  | 3012 | C | A | | Missense |
|  | 3540 | A | T | | Missense |
|  | 3563 | A | G | | Synonymous |
| **HOG pathway** | | | | | |
| *SHO1* | 460 | G | T | | Missense |
|  | 798 | C | T | | Synonymous |
| *SSK1* | 545 | G | A | | Synonymous |
|  | 1131 | G | A | | Missense |
| *SKN7* | 71 | C | A | | Missense |
| *SSK2* | 353 | C | G | | Synonymous |
|  | 362 | G | T | | Synonymous |
|  | 368 | A | T | | Synonymous |
|  | 467 | C | T | | Synonymous |
|  | 542 | A | G | | Synonymous |
|  | 764 | C | T | | Synonymous |
|  | 777 | C | T | | Synonymous |
|  | 1034 | A | G | | Synonymous |
|  | 1112 | T | G | | Missense |
|  | 1401 | G | A | | Missense |
|  | 1724 | A | G | | Missense |
|  | 1934 | A | G | | Synonymous |
|  | 1955 | A | G | | Synonymous |
|  | 2136 | C | T | | Synonymous |
|  | 2177 | G | T | | Synonymous |
|  | 2240 | T | C | | Synonymous |
|  | 2381 | G | A | | Synonymous |
|  | 2540 | A | G | | Synonymous |
|  | 2853 | A | T | | Missense |
|  | 2999 | C | T | | Synonymous |
|  | 3713 | C | T | | Synonymous |
|  | 3824 | T | C | | Synonymous |
|  | 3878 | T | A | | Synonymous |
| *SSK22* | 749 | A | G | | Synonymous |
|  | 946 | G | T | | Missense |
|  | 1004 | A | G | | Synonymous |
|  | 1265 | C | T | | Synonymous |
|  | 1278 | G | T | | Missense |
|  | 1448 | C | T | | Synonymous |
|  | 1482 | C | T | | Missense |
|  | 1523 | C | T | | Synonymous |
|  | 1787 | C | T | | Synonymous |
|  | 1871 | T | C | | Synonymous |
|  | 1973 | A | G | | Synonymous |
|  | 3660 | A | G | | Missense |
|  | 3665 | G | C | | Synonymous |
|  | 3695 | T | C | | Synonymous |
| *PBS2* | 29 | A | C | | Synonymous |
|  | 605 | C | T | | Synonymous |
|  | 1532 | A | G | | Synonymous |
|  | 1628 | G | A | | Synonymous |
|  | 1745 | A | G | | Synonymous |
|  | 1925 | G | A | | Synonymous |
| *HOG1* | 168 | TTGAGCCTGAGCCT  GAGCCTGAGCCTG | TTGAGCCTGAG  CCTGAGCCTG | | Conservative inframe deletion |
|  | 396 | A | G | | Synonymous |
|  | 642 | G | A | | Synonymous |
|  | 681 | G | A | | Synonymous |
|  | 1059 | A | T | | Synonymous |
|  | 1260 | C | T | | Synonymous |
| *PTP2* | 553 | T | C | | Missense |
|  | 675 | G | A | | Missense |
|  | 1595 | T | C | | Synonymous |
| *PTP3* | 589 | GCATCATCATCATC  ATCATCATCATCATCA  TCATCATCATCATCATCA  TCATCATCA | GCATCATCATCA  TCATCATCATCA  TCATCATCATCA  TCATCATCATCATCATCA | | Conservative inframe deletion |
|  | 2667 | G | A | | Synonymous |
| *SLT2* | 1123 | TACAGCAACAGC  AACAGCAACAGC  AACAGCAACAGCA | TACAGCAACAGC  AACAGCAACAGC  AACAGCAACAGC  AACAGCAACAGCA | | Disruptive inframe deletion |
| **CWI pathway** | | | | | |
| *WSC2* | 107 | C | T | Synonymous | |
|  | 518 | T | C | Synonymous | |
| *WSC3* | \| 960 \| \| --- \| | GGAGGTAGTGGA  AGAGGTAGTGGA  AGAGGTAGTGGA  AGAGGTAGT | GGAGGTAGTGGA  AGAGGTAGTGGA  AGAGGTAGT | Conservative inframe deletion | |
|  | \| 993 \| \| --- \| | G | A | Synonymous | |
|  | 996 | A | G | Synonymous | |
| *PKC1* | 149 | C | T | Upstream | |
|  | 223 | TAAAAAAA | TAAAAA | Upstream | |
|  | 306 | A | G | Upstream | |
|  | 1912 | C | T | Missense | |
|  | 1919 | C | T | Missense | |
|  | 1957 | C | T | Missense | |
|  | 3579 | A | G | Synonymous | |
| *SLT2* | 1123 | TACAGCAACAGC  AACAGCAACAGCA  ACAGCAACAGCA | TACAGCAACAGC  AACAGCAACAGC  AACAGCAACAGC  AACAGCAACAGCA | Disruptive inframe deletion | |
| *RLM1* | 272 | C | T | Synonymous | |
|  | 297 | T | C | Synonymous | |
|  | 344 | G | A | Synonymous | |
|  | 392 | T | C | Synonymous | |
|  | 986 | A | G | Synonymous | |
|  | 1179 | A | G | Missense | |
|  | 1560 | A | G | Missense | |
|  | 1690 | G | A | Missense | |
|  | 1706 | TAACAACAACAA  CAACAATAACAA  CAACAACA | TAACAACAACAA  CAATAACAACAA  CAACA | Disruptive inframe deletion | |
|  | 1932 | C | T | Synonymous | |
| *SWI4* | 93 | C | T | Synonymous | |
|  | 244 | A | G | Missense | |
| **Cell wall biogenesis** | | | | | |
| *SLT2* | 1123 | TACAGCAACAGC  AACAGCAACAGC  AACAGCAACAGCA | TACAGCAACAGC  AACAGCAACAGC  AACAGCAACAGC  AACAGCAACAGCA | Disruptive inframe deletion | |
| *HSP150* | 369 | G | T | Synonymous | |
|  | 603 | G | A | Synonymous | |
|  | 731 | T | C | Missense | |
|  | 741 | A | G | Synonymous | |
|  | 744 | G | A | Synonymous | |
|  | 1182 | G | A | Synonymous | |
| *CHS1* | 62 | ATTTTTTTTTTTT | ATTTTTTTTTTT | Frameshift | |
|  | 252 | A | G | Synonymous | |
|  | 519 | G | A | Synonymous | |
|  | 666 | A | G | Synonymous | |
|  | 1434 | G | A | Synonymous | |
|  | 1467 | T | C | Synonymous | |
|  | 1619 | A | G | Synonymous | |
| *ACF2* | 330 | C | T | Synonymous | |
|  | 590 | T | A | Missense | |
|  | 591 | T | A | Missense | |
|  | 1683 | A | G | Missense | |
|  | 1769 | C | T | Synonymous | |
|  | 1775 | C | T | Synonymous | |
|  | 1910 | A | G | Synonymous | |
| *PIR3* | 413 | G | A | Synonymous | |
|  | 461 | C | T | Synonymous | |
|  | 491 | C | T | Synonymous | |
|  | 497 | T | C | Synonymous | |
|  | 590 | A | G | Synonymous | |
|  | 596 | T | C | Synonymous | |
|  | 599 | C | T | Synonymous | |
|  | 623 | T | C | Synonymous | |
|  | 641 | C | T | Synonymous | |
|  | 677 | C | T | Synonymous | |
|  | 680 | C | T | Synonymous | |
|  | 704 | C | T | Synonymous | |
|  | 731 | C | T | Synonymous | |
| *SED1* | 141 | A | G | Synonymous | |
|  | 378 | G | T | Synonymous | |
|  | 393 | G | A | Synonymous | |
|  | 396 | G | A | Synonymous | |
|  | 420 | T | A | Synonymous | |
|  | 438 | G | A | Synonymous | |
|  | 589 | A | G | Missense | |
|  | 599 | T | A | Missense | |
|  | 688 | G | C | Missense | |
|  | 754 | G | C | Missense | |
|  | 865 | C | T | Missense | |
| *SPI1* | 188 | C | T | Synonymous | |
|  | 275 | C | T | Synonymous | |
|  | 382 | T | G | Missense | |
|  | 404 | G | C | Synonymous | |
| *TIR3* | 74 | G | A | Synonymous | |
|  | 215 | C | T | Synonymous | |
|  | 369 | T | A | Missense | |
|  | 473 | T | A | Synonymous | |
| *TIP1* | 462 | G | A | Synonymous | |
|  | 528 | A | T | Synonymous | |
|  | 645 | A | G | Synonymous | |
| *KRE5* | 398 | C | T | Synonymous | |
|  | 647 | C | T | Synonymous | |
|  | 1150 | G | A | Missense | |
|  | 2177 | G | A | Synonymous | |
|  | 2214 | G | A | Missense | |
|  | 2240 | T | C | Synonymous | |
|  | 2760 | C | T | Synonymous | |
|  | 2855 | A | G | Synonymous | |
|  | 2969 | A | C | Synonymous | |
|  | 3189 | T | C | Synonymous | |
| **Arginine biosynthesis** | | | | | |
| *ARG5,6* | 2117 | C | G | Synonymous | |
|  | 2255 | G | A | Synonymous | |
|  | 2406 | A | G | Missense | |
|  | 2459 | C | T | Synonymous | |
| **Heat shock protein** | | | | | |
| *HSP60* | 54 | T | C | Synonymous | |
| *HSP104* | 209 | A | G | Synonymous | |
|  | 796 | T | C | Missense | |
